# Facile Construction and Fabrication of a Superhydrophobic and Super Oleophilic Stainless Steel Mesh for Separation of Water and Oil

**DOI:** 10.3390/nano12101661

**Published:** 2022-05-13

**Authors:** Yinyu Sun, Zhongcheng Ke, Caiyun Shen, Qing Wei, Ruikang Sun, Wei Yang, Zihan Yin

**Affiliations:** School of Chemistry and Chemical Engineering, Huangshan University, Huangshan 245021, China; yysun_hsu@163.com (Y.S.); ascy8843@163.com (C.S.); wq19105591009@163.com (Q.W.); sunruikangjnu@163.com (R.S.); myxhjsn996320687@163.com (W.Y.); yzh20030201@163.com (Z.Y.)

**Keywords:** stainless steel mesh, Fe_2_O_3_ nanoclusters, octadecyltrimethoxysilane, oil–water separation

## Abstract

The fluoride-free fabrication of superhydrophobic materials for the separation of oil/water mixtures has received widespread attention because of frequent offshore oil exploration and chemical leakage. In recent years, oil/water separation materials, based on metal meshes, have drawn much attention, with significant advantages in terms of their high mechanical strength, easy availability, and long durability. However, it is still challenging to prepare superhydrophobic metal meshes with high-separation capacity, low costs, and high recyclability for dealing with oil–water separation. In this work, a superhydrophobic and super oleophilic stainless steel mesh (SSM) was successfully prepared by anchoring Fe_2_O_3_ nanoclusters (Fe_2_O_3_-NCs) on SSM via the in-situ flame synthesis method and followed by further modification with octadecyltrimethoxysilane (OTS). The as-prepared SSM with Fe_2_O_3_-NCs and OTS (OTS@Fe_2_O_3_-NCs@SSM) was confirmed by a field emission scanning electron microscope (FESEM), transmission electron microscope (TEM), energy dispersive spectrometer (EDS), X-ray photoelectron spectrometer (XPS), and X-ray diffractometer (XRD). The oil–water separation capacity of the sample was also measured. The results show that the interlaced and dense Fe_2_O_3_-NCs, composed of Fe_2_O_3_ nanoparticles, were uniformly coated on the surface of the SSM after the immerging-burning process. Additionally, a compact self-assembled OTS layer with low surface energy is coated on the surface of Fe_2_O_3_-NCs@SSM, leading to the formation of OTS@Fe_2_O_3_-NCs@SSM. The prepared OTS@Fe_2_O_3_-NCs@SSM shows excellent superhydrophobicity, with a water static contact angle of 151.3°. The separation efficiencies of OTS@Fe_2_O_3_-NCs@SSM for the mixtures of oil/water are all above 98.5%, except for corn oil/water (97.5%) because of its high viscosity. Moreover, the modified SSM exhibits excellent stability and recyclability. This work provides a facile approach for the preparation of superhydrophobic and super oleophilic metal meshes, which will lead to advancements in their large-scale applications on separating oil/water mixtures.

## 1. Introduction

The cost-effective separation strategies of oil/water mixtures have become a hot topic due to the frequent occurrences of water pollution issues, such as organic wastewater emissions, chemical leaks, and crude oil spills [1,2,3,4]. To date, a series of materials, including carbon aerogels [5,6,7,8], cotton fabrics [9,10,11,12], foams [13,14,15], and sponges [16,17,18,19], have been explored, owing to the excellent hydrophobicity and high separation capacity. However, some materials are difficult to use in large-scale applications because of the complicated and high-cost separation process, secondary contamination, or poor recyclability. From the perspective of application, it is necessary to develop a more efficient, facile, and low-cost method to achieve the separation of water and oil.

Over the past few years, oil/water separation materials, based on metal meshes [20,21,22], especially stainless steel meshes (SSM) [23,24,25,26], have drawn much attention, with significant advantages in terms of their high mechanical strength, easy availability, and long durability. The preparation of superhydrophobic mesh is mainly based on surface modification by changing the chemical composition or the microscopic geometry of the surface [27,28,29,30]. For example, Jiang et al. first prepared a superhydrophobic mesh with high oil–water separation efficiency via surface modification with polytetrafluoroethylene [31]. Chen et al. provided a facile approach for the preparation of oil–water separation material via the coating of the reduced graphene oxide on mesh [32]. Fu and co-workers prepared a robust superhydrophobic and super oleophilic material by loading hierarchical-layer nanospheres on the surface of metal mesh [33]. Liang et al. developed a co-precipitation strategy to prepare superhydrophobic materials by growing hierarchical micro-nanostructures of CeO_2_ on the Cu mesh, followed by further modification with stearic acid [21]. Wang et al. reported that a superhydrophobic mesh was prepared via the coating of micro/nanostructured Co(OH)_2_ on the mesh, followed by modification with hexadecyltrimethoxysilane [27]. Based on the aforementioned previous research, the rough surface caused by the loading of nanoparticles can greatly increase the surface area of the substrate. Furthermore, the superhydrophobic surface is usually achieved by surface modification with a low-surface-energy material. Nevertheless, most methods of preparing the oil/water separation material are usually costly, such as expensive equipment or devices, complex synthesis steps, toxic reagents, or by-products. Therefore, it is necessary to develop a facile strategy to prepare superhydrophobic SSM with high separation capacity, low costs, and high recyclability for dealing with oil/water separation.

In this work, an SSM with a superhydrophobic and super oleophilic surface was successfully prepared by anchoring Fe_2_O_3_ nanoclusters (Fe_2_O_3_-NCs) by the in-situ flame synthesis method and then modified with octadecyltrimethoxysilane (OTS). The SSM with OTS and Fe_2_O_3_-NCs (OTS@Fe_2_O_3_-NCs@SSM) was characterized with a field emission scanning electron microscope (FESEM), energy dispersive X-ray spectrometer (EDS), X-ray photoelectron spectrometer (XPS), and X-ray diffractometer (XRD). Additionally, the oil–water separation capacity and recyclability of the samples were also examined.

## 2. Materials and Methods

### 2.1. Materials

Ferric acetylacetonate (C_15_H_21_FeO_6_, 99%), absolute ethanol, and OTS were purchased from Macklin biochemical technology Co., Ltd., China (Shanghai, China). The commercial SSM of 400 meshes was purchased from Taobao (Changzhou, China). The SSM with a size of 5 × 5 cm was ultrasonically cleaned in ethanol for 1 h and then dried at 80 °C in air for 5 h. Distilled water was used in all the experiments. 

### 2.2. Preparation of OTS@Fe_2_O_3_-NCs@SSM

Firstly, SSM was dipped into a solution containing 100 mL of ethanol and 2.2 g of C_15_H_21_FeO_6_ for 5 s to get SSM filled with the solution and followed by burning in air. This process was repeated 40 times to ensure the loading of Fe_2_O_3_-NCs on SSM (Fe_2_O_3_-NCs@SSM). The as-obtained Fe_2_O_3_-NCs@SSM was cleaned with deionized water and dried at 80 °C in air for 3 h. Secondly, 0.5 mL of OTS was ultrasonically dissolved in 400 mL of distilled water for 1 h. Subsequently, Fe_2_O_3_-NCs@SSM was added into the aforementioned aqueous emulsion and left for 24 h. Finally, the as-obtained OTS@Fe_2_O_3_-NCs@SSM was cleaned with deionized water and dried at 80 °C in air for 5 h. 

### 2.3. Physical Characterizations

The morphologies were observed using FESEM (Gemini 500, Zeiss, Oberkochen, Germany) and TEM (Tecnai G^2^ F20, FEI, Ames, IA, USA). The surface elemental composition and distribution were determined by EDS (X-Max^N^ 80, Oxford, UK) and XPS (ESCALAB 250Xi, Thermo Fisher, Waltham, MA, USA). X-ray diffraction patterns were obtained by XRD equipped with a Cu Kα radiation source (D8 Advance, Bruker, Karlsruher, Germany). The water static contact angle was measured at room temperature via a contact angle meter (DSA100, KRUSS, Hamburg, Germany). Zeta potential values were obtained using a solid zeta potential instrument (SurPass, Anton Paar, Graz, Austria) and nanoparticle size potentiometer (Nano ZS90, Malvern, Malvern, UK).

### 2.4. Oil–Water Separation and Recyclability Tests

In the oil/water separation test, the immiscible mixtures of water and oil or organic solvents in a 1:1 volume ratio were used. The mixtures were poured onto OTS@Fe_2_O_3_-NCs@SSM, which was fixed in the homemade device. The oil–water separation efficiency T was calculated by (V_2_/V_1_) × 100, where V_1_ and V_2_ were the volume of water before and after separation, respectively. In the recyclability test, the separation process of the samples was repeated. After each cycle, the used samples were cleaned with ethanol and dried at 80 °C in air for 3 h.

## 3. Results and Discussion

### 3.1. Characterization

**Figure 1:**
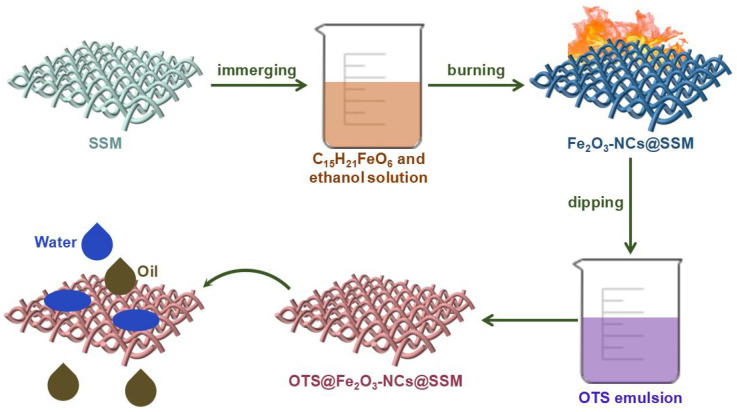
Schematic illustration of the preparation of OTS@Fe_2_O_3_-NCs@SSM.

Figure 1 presents the schematic illustration of the controllable constructing process of OTS@Fe_2_O_3_-NCs@SSM via a simple and facile two-step strategy. Firstly, SSM wetted by the solution containing ethanol and C_15_H_21_FeO_6_ was taken out and burned in air. In this immerging-burning process, absolute ethanol was not only used as a solvent to dissolve C_15_H_21_FeO_6_, but also made C_15_H_21_FeO_6_ sufficient for burning in air. In the meantime, hydrocarbon moieties and Fe atoms decomposed by C_15_H_21_FeO_6_ at high temperatures acted as a source of carbon atoms and catalyst, respectively [34,35]. The hydrocarbon moieties were coated on Fe atoms to form an Fe–C bond [36]. Fe_2_O_3_-NCs nanoparticles were constantly deposited on the carbon precipitates to grow Fe_2_O_3_-NCs on the surface of SSM after repeating 40 times for the immerging-burning process. The immobilization of Fe_2_O_3_-NCs on the surface of SSM is not only beneficial to enhance the surface roughness of SSM, but can also effectively improve the adhesion between hydrophobic components and SSM. Secondly, Fe_2_O_3_-NCs@SSM was added into the OTS aqueous emulsion, leading to the formation of OTS@Fe_2_O_3_-NCs@SSM. In particular, it needs to be mentioned that the growing of Fe_2_O_3_-NCs on SSM after immerging-burning treatment is beneficial to the coating of OTS on the surface of Fe_2_O_3_-NCs@SSM, which will enhance the superhydrophobic property of the material. Additionally, OTS can be used to grow a compact and self-assembled monolayer with low surface energy on the surface of Fe_2_O_3_-NCs@SSM, leading to an improvement in the hydrophobicity of the sample [37,38].

**Figure 2:**
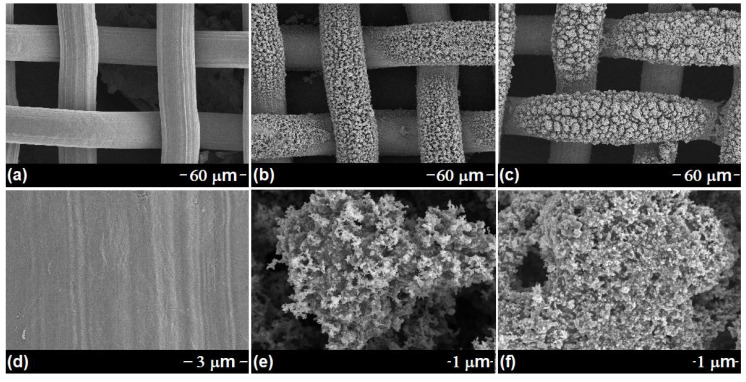
FESEM images of SSM (**a**,**d**), Fe_2_O_3_-NCs@SSM (**b**,**e**), OTS@Fe_2_O_3_-NCs@SSM (**c**,**f**).

**Figure 3:**
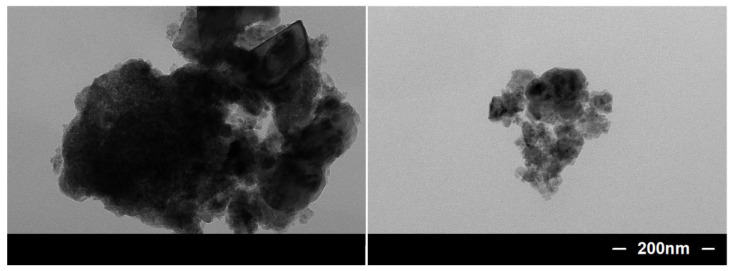
TEM image of Fe_2_O_3_-NCs.

The surface morphology of samples was demonstrated by FESEM and TEM images. As shown in Figure 2a,d, the typical FESEM images of the untreated SSM exhibit a smooth surface, with a diameter of ca. 34 μm, which is used as the substrate to adsorb C_15_H_21_FeO_6_. After the dipping–burning process, the interlaced and dense Fe_2_O_3_-NCs are uniformly coated on the surface of the SSM, with a diameter of ca. 40 μm (Figure 2b,e). The thickness of the Fe_2_O_3_-NCs layer was found to be ca. 3 µm, based on the difference in diameter between SSM and Fe_2_O_3_-NCs@SSM. The TEM image (Figure 3) clearly confirms that Fe_2_O_3_-NCs are composed of Fe_2_O_3_ nanoparticles. It is notable that Fe_2_O_3_-NCs not only provides the larger surface area for the loading of OTS, but also effectively enhances the adhesion between OTS and SSM. After modification, a dense self-assembled OTS layer is coated on the surface of Fe_2_O_3_-NCs@SSM, leading to the formation of OTS@Fe_2_O_3_-NCs@SSM (Figure 2c,f).

**Figure 4:**
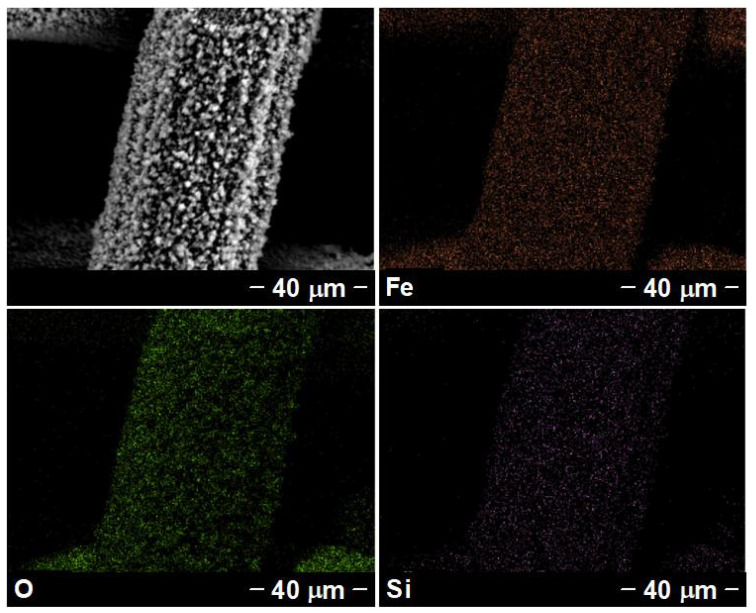
Images of OTS@Fe_2_O_3_-NCs@SSM on EDS elemental mappings.

**Figure 5:**
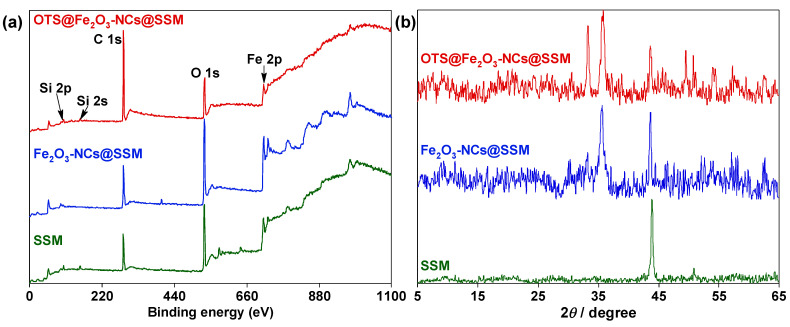
(**a**) XPS analysis of SSM, Fe_2_O_3_-NCs@SSM, and OTS@Fe_2_O_3_-NCs@SSM. (**b**) XRD patterns of SSM, Fe_2_O_3_-NCs@SSM, and OTS@Fe_2_O_3_-NCs-NCs@SSM.

The chemical compositions of samples were tested by EDS and XPS. The SEM-based EDS elemental analysis results (Figures S1–S3) revealed that the elemental content of O on Fe_2_O_3_-NCs@SSM was obviously increased, compared with SSM, due to the formation of Fe_2_O_3_-NCs on the surface of SSM after the immerging-burning process. It is worth noting that the proportion of Si element on SSM and Fe_2_O_3_-NCs@SSM are 0.31 and 0%, respectively. These results suggest that the Si element was undetected on the surface of Fe_2_O_3_-NCs@SSM because of the interlaced and dense Fe_2_O_3_-NCs coated on SSM. As described in Figure S3 and Figure 4, the elemental distribution of Si components on OTS@Fe_2_O_3_-NCs@SSM can verify that the hydrophobic components of OTS are successfully coated on the substrate. As shown in Figure 5a, the surface XPS results of SSM verify the existence of C (284.6 eV), O (530.6 eV), and Fe (711.2 eV) elements [30]. In addition, the Si 2s and Si 2p signal peaks are detected on the surface of SSM, which is consistent with the EDS results (Figure S1). The absence of Si 2s peak in the XPS results of Fe_2_O_3_-NCs@SSM may be caused by the coating of Fe_2_O_3_-NCs on SSM, which agrees well with the EDS results shown in Figure S2. The Si 2s and Si 2p peaks are present in the XPS results of OTS@Fe_2_O_3_-NCs@SSM, which originated from OTS [39]. These XPS results can further confirm the successful preparation of Fe_2_O_3_-NCs and OTS on the surface of SSM.

As shown in Figure 5b, the crystal phases of SSM, Fe_2_O_3_-NCs@SSM, and OTS@Fe_2_O_3_-NCs@SSM were analyzed by XRD patterns. The XRD patterns of the samples have two characteristic peaks at 44.0 and 51.1°, which are assigned to the (111) and (200) lattice planes of SSM, respectively [30]. In addition, the diffraction patterns of Fe_2_O_3_-NCs@SSM exhibit four characteristic peaks, corresponding to the (104), (110), (024), and (214) lattice planes of Fe_2_O_3_ nanoparticles at 33.2, 35.6, 49.5, and 62.4°, respectively [37]. This result can further confirm the successful preparation of Fe_2_O_3_-NCs on the surface of SSM. No obvious characteristic diffraction peaks for OTS can be detected due to its poor crystallinity. As can be seen in Table S1, the zeta potentials of the samples were measured. The charge of SSM is −63.25 mV in 1 mM KCl aqueous solution at pH = 7, which is consistent with previous studies [40,41]. After the immerging-burning process, the surface charge of Fe_2_O_3_-NCs@SSM increases to −45.09 mV due to the coating of Fe_2_O_3_-NCs with a zeta potential value of −14.17 mV. Moreover, the zeta potential of OTS@Fe_2_O_3_-NCs@SSM is still negatively charged with a value of −27.83 mV, which can be attributed to the further loading of OTS, with a zeta potential value of −26.10 mV. These results suggest that Fe_2_O_3_-NCs and OTS are successfully immobilized on the surface of SSM. 

### 3.2. Superhydrophobic Performance

**Figure 6:**
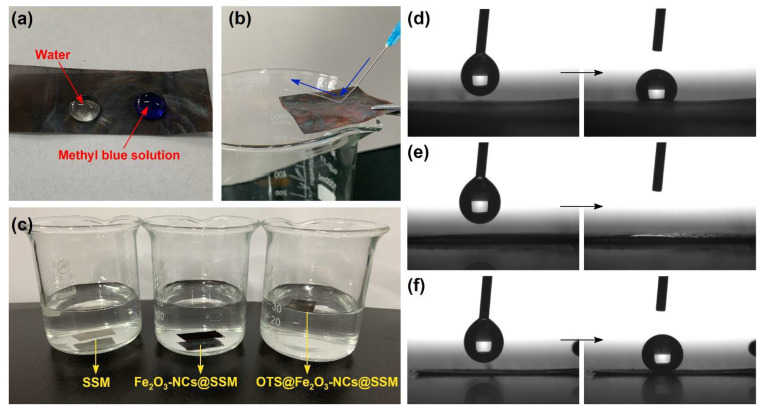
(**a**) Water and methyl blue droplets on the top of OTS@Fe_2_O_3_-NCs@SSM. (**b**) The water column reflects image on the surface of OTS@Fe_2_O_3_-NCs @SSM. (**c**) The floating test on water of the samples. (**d**–**f**) The water static contact-angle measurement of SSM, Fe_2_O_3_-NCs@SSM, and OTS@Fe_2_O_3_-NCs @SSM.

The surface hydrophobicity of the sample is very important to support potential practical applications. As depicted in Figure 6a, water and methyl blue solution droplets were prevented from penetrating through OTS@Fe_2_O_3_-NCs@SSM, exhibiting excellent hydrophobic properties. Interestingly, a water column ejected from a syringe and bouncing off the superhydrophobic surface of OTS@Fe_2_O_3_-NCs@SSM, indicating that the modified SSM has an ultralow contact-angle hysteresis (Figure 6b). To further evaluate the hydrophobicity of the samples, the floating test and the water static contact-angle test were carried out. As shown in Figure 6c, both SSM and Fe_2_O_3_-NCs@SSM sink to the bottom of water. However, OTS@Fe_2_O_3_-NCs@SSM can float on water and pick it up without water droplets. This result indicates that OTS@Fe_2_O_3_-NCs@SSM has better hydrophobicity than SSM and Fe_2_O_3_-NCs@SSM. As can be seen in Figure 6d–f, the water static contact angle of the samples was observed. The original SSM exhibits a hydrophilic property with a water static contact angle of ca. 116.5° (Figure 6d). The water static contact angle of Fe_2_O_3_-NCs@SSM is ca. 0° (Figure 6e). This result suggests that the hydrophilic component of Fe_2_O_3_-NCs changes the surface hydrophobicity of SSM. After modification, OTS@Fe_2_O_3_-NCs@SSM shows extreme superhydrophobic surface with a water static contact angle of 151.3^o^ because of the uniform coating of the hydrophobic component of OTS on Fe_2_O_3_-NCs@SSM (Figure 6f). The preparation of superhydrophobic SSM can be explained by changing the microscopic geometry and the surface energy. Firstly, the rough surface caused by the formation of Fe_2_O_3_-NCs can retain more air beneath the water droplets, effectively reducing the solid–liquid contact area. Furthermore, OTS, a typical low-surface-energy material, is used to modify the surface property of Fe_2_O_3_-NCs@SSM, leading to enhanced hydrophobicity.

**Figure 7:**
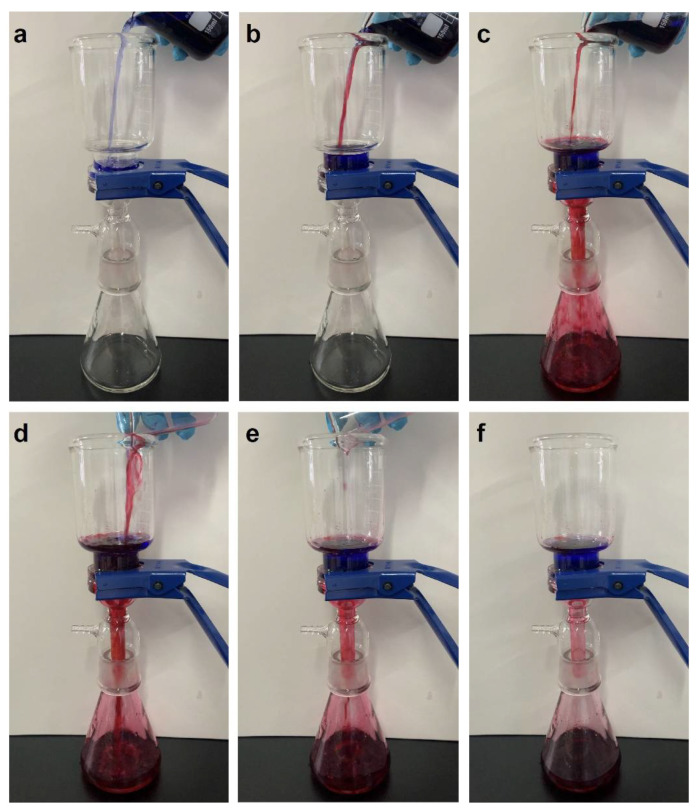
(**a**–**f**) Oil–water separation process of OTS@Fe_2_O_3_-NCs@SSM (water dyed with methyl blue and dichloromethane dyed with Oil red O).

To ensure a better evaluation for the superhydrophobic behavior of the samples, the oil–water separation performance was tested. As shown in Figure 7, dichloromethane and water were dyed with oil red O and methyl blue, respectively. Subsequently, the mixtures were poured into the homemade gravity-driven separation device through OTS@Fe_2_O_3_-NCs@SSM. It was found that dichloromethane can quickly permeate through OTS@Fe_2_O_3_-NCs@SSM through the driving force of gravity and water retained on the top side of the modified mesh. The prepared OTS@Fe_2_O_3_-NCs@SSM has excellent performance to separate the oil/water mixtures due to the superhydrophobic and super oleophilic surfaces.

**Figure 8:**
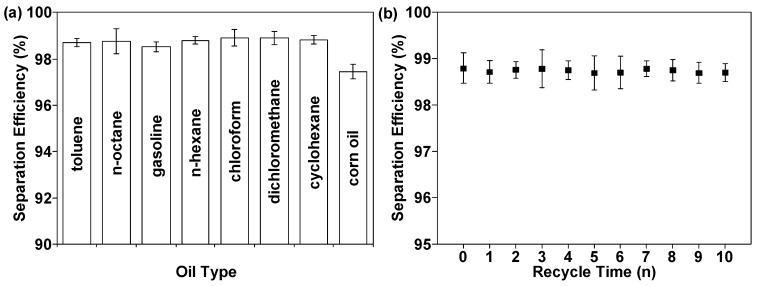
(**a**) Oil/water separation efficiencies of OTS@Fe_2_O_3_-NCs@SSM for oil or organic solvents. (**b**) Separation efficiency for the n-hexane/water mixture after 10 cycles.

As depicted in Figure 8a, the oil–water separation efficiency was evaluated by the homemade separation device. The separation efficiencies of OTS@Fe_2_O_3_-NCs@SSM for the mixtures of petroleum toluene/water, n-octane/water, gasoline/water, n-hexane/water, chloroform/water, dichloromethane/water, and cyclohexane/water were all above 98.5%, except for corn oil/water (97.5%), because of its high viscosity. These data suggest that the prepared superhydrophobic SSM can be used as an ideal material to efficiently separate an oil/water mixture. As can be seen from the data in Table 1, the modified SSM shows higher separation efficiency than many previously reported meshes [22,24,33,42,43,44,45,46]. Although the separation efficiency of OTS@Fe_2_O_3_-NCs@SSM is still lower than that of those reported materials [21,27,32], the preparation method of OTS@Fe_2_O_3_-NCs@SSM is more facile, time saving, and economical. Therefore, OTS@Fe_2_O_3_-NCs@SSM is a cost-effective and promising material for oil/water separation. In addition, the oil–water separation materials should be easily cleaned and recycled in terms of the practical applications. The recycled experiments of the superhydrophobic and super oleophilic OTS@Fe_2_O_3_-NCs@SSM were also investigated (Figure 8b). The separation efficiency of the samples for the n-hexane/water mixture could be maintained above 98.6% after 10 cycles of separation, indicating the excellent reusability of the samples. In addition, data analysis of group 10 and group 0 shows that the *p* value is greater than 0.05, suggesting that the separation efficiency of the two groups is not significantly different. This result suggests that the immobilization of Fe_2_O_3_-NCs on the surface of SSM can effectively improve the adhesion between hydrophobic components and SSM and enhance the reusability of the material.

## 4. Conclusions

In summary, a robust and stable superhydrophobic and super oleophilic SSM was fabricated successfully by anchoring Fe_2_O_3_-NCs on SSM via the in-situ flame synthesis method and followed by further modification with OTS. The characterization results indicate that the preparation of Fe_2_O_3_-NCs, composed of Fe_2_O_3_ nanoparticles on the surface of SSM, is not only beneficial to enhance the surface roughness of SSM, but can also effectively improve the adhesion between hydrophobic components and SSM. In addition, the coating of OTS with low surface energy endows SSM with excellent superhydrophobicity, as well as super oleophilicity, with a water static contact angle of 151.3°. Moreover, the prepared OTS@Fe_2_O_3_-NCs@SSM exhibits excellent oil–water separation efficiency for a series of oil and water mixtures. It is important to note that the modified SSM shows good stability and reusability after 10 cycles. Therefore, this work provides an intriguing methodology for coating nanostructures on metal substrates, which will lead to a new perspective on separating oil/water mixtures.

## Figures and Tables

**Table 1:** Comparison of various meshes for oil/water separation.

Matrix	Modified Materials	Method	Separation Efficiency (%)	Ref.
wire mesh	Graphene oxide	O_2_ plasma and thermal annealing	>98	[32]
Cu mesh	1-dodecanethiol	Immersion and modification	>96	[22]
Cu Mesh	Lauric acid	Electrodeposition and modification	>93	[40]
Cu mesh	Na_2_SiO_3_ + Al_2_O_3_	Self-assemble	>95	[41]
Cu mesh	Stearic acid	Co-precipitation and modification	>99	[21]
SSM	Phytic acid and vinyltriethoxysilane	Immersion	>90	[33]
SSM	Natural flake mica	Hydrothermal synthesis and electrodeposition	>90	[42]
SSM	—	Laser ablation	>96	[24]
SSM	Hexadecyltrimethoxysilane	Pulse electrodeposition and modification	>99	[27]
SSM	TiO_2_ nanofibers	Hydrothermal synthesis and spray deposition	>90	[43]
SSM	Glass particles	Laser texturing	>96	[44]
SSM	Fe_2_O_3_-NCs and OTS	Flame synthesis and modification	>97	This work

## Data Availability

The data presented in this study are available on request from the corresponding author.

## Supplementary Materials

The following supporting information can be downloaded at: https://www.mdpi.com/article/10.3390/nano12101661/s1, Figure S1: Images of SSM on EDS elemental analysis; Figure S2: Images of Fe_2_O_3_-NCs@SSM on EDS elemental analysis.; Figure S3: Images of OTS@Fe_2_O_3_-NCs@SSM on EDS elemental analysis; Table S1: Zeta potential values of the samples.
