# Supplementary material for: Facile Construction and Fabrication of a Superhydrophobic and Super Oleophilic Stainless Steel Mesh for Separation of Water and Oil
Source: Nanomaterials (Basel). 2022 May 13;12(10):1661. doi: 10.3390/nano12101661 (PMC9147946; doi:10.3390/nano12101661)
Supplement: Supplementary file 1 [file nanomaterials-12-01661-s001.zip › nanomaterials-1720174-supplementary.pdf]

nanomaterials

Supplementary Materials

Facile construction and fabrication of a superhydrophobic and super oleophilic stainless steel mesh for separation of water and oil

Yinyu Sun, Zhongcheng Ke*, Caiyun Shen, Qing Wei, Ruikang Sun, Wei Yang and Zihan Yin

School of Chemistry and Chemical Engineering, Huangshan University, Huangshan 245021, Anhui, China; yysun_hsu@163.com

* Correspondence: xiaoke1020@126.com; Tel.: +86-13955997920

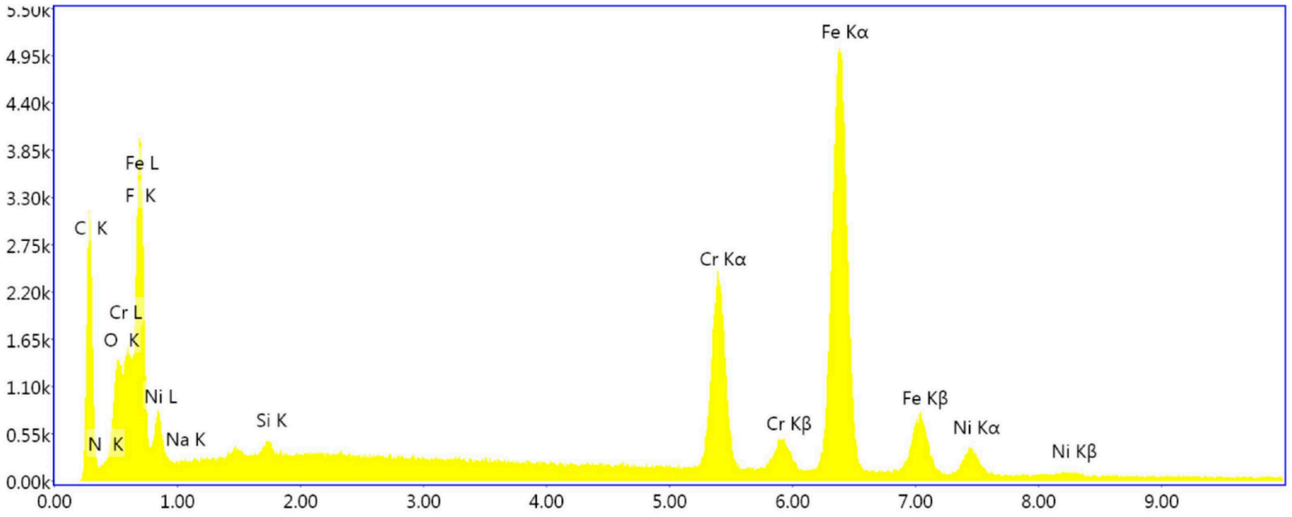

Figure S1. Images of SSM on EDS elemental analysis.

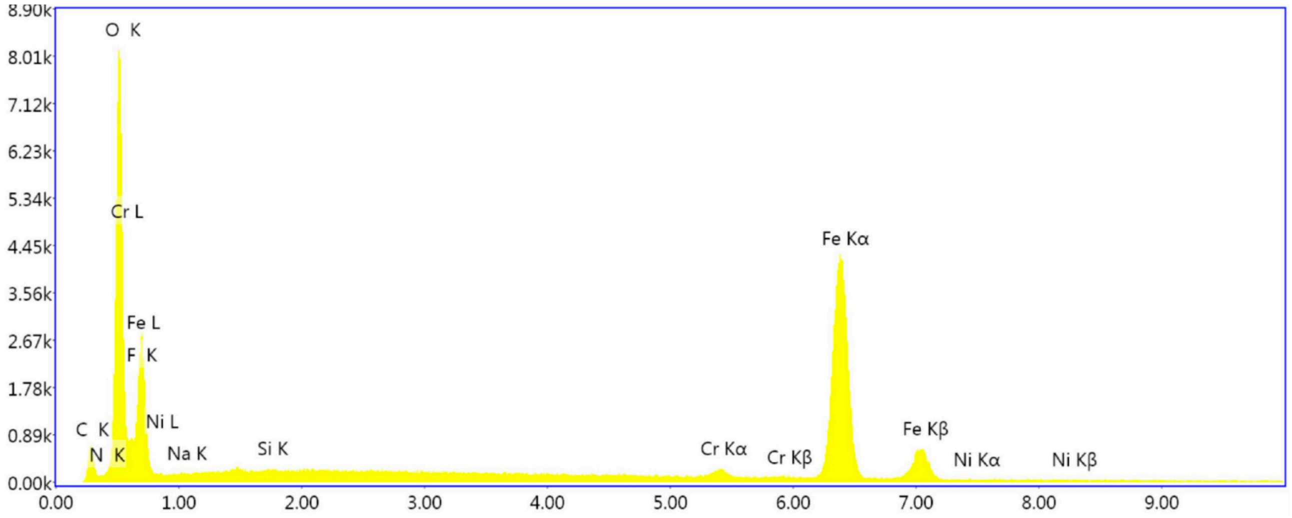

Figure S2. Images of Fe_2O_3-NCs@SSM on EDS elemental analysis.

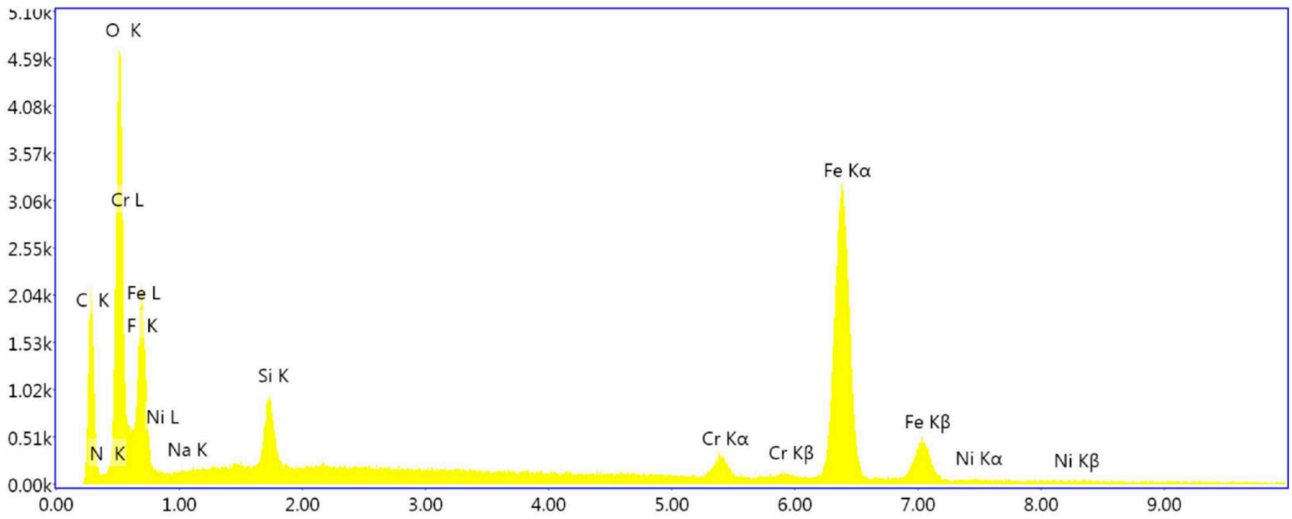

Figure S3. Images of OTS@Fe_2O_3-NCs@SSM on EDS elemental analysis.

Table 1. Zeta potential values of the samples.

Sample	Zeta potential (mv)	Stdev (mv)	Test device
SSM	-63.25	5.01	SurPass, Anton Paar
Fe_2O_3-NCs@SSM	-45.09	5.71	SurPass, Anton Paar
OTS@Fe_2O_3-NCs@SSM	-27.83	6.81	SurPass, Anton Paar
Fe_2O_3-NCs	-14.17	0.48	Nano ZS90, Malvern
OTS	-26.10	0.36	Nano ZS90, Malvern
